# Supplementary material for: Hydroquinone redox mediator enhances the photovoltaic performances of chlorophyll-based bio-inspired solar cells
Source: Commun Chem. 2021 Aug 11;4:118. doi: 10.1038/s42004-021-00556-5 (PMC9814249; doi:10.1038/s42004-021-00556-5)
Supplement: Supplementary file 1 — Supplementary Information [file 42004_2021_556_MOESM1_ESM.pdf]

Hydroquinone redox mediator enhances the photovoltaic performance of chlorophyll-based bio-inspired solar cell

Shengnan Duan [a,b,c], Chiasa Uragami [b], Kota Horiuchi [b], Kazuki Hino [b], Xiao-Feng Wang [a*], Shin-ichi Sasaki [d,e], Hitoshi Tamiaki [e], Hideki Hashimoto [b*]

[a] Key Laboratory of Physics and Technology for Advanced Batteries (Ministry of Education), College of Physics, Jilin University, Changchun, 130012, P. R. China

[b] Department of Applied Chemistry for Environment, Faculty of Science and Technology, Kwansei Gakuen University, Sanda, Hyogo, 669-1337, Japan

[c] School of Science, Chongqing University of Posts and Telecommunications, Chongqing, 400065, P. R. China

[d] Nagahama Institute of Bio-Science and Technology, Nagahama, Shiga, 526-0829, Japan

[e] Graduate School of Life Sciences, Ritsumeikan University, Kusatsu, Shiga, 525-8577, Japan

Corresponding Authors:

Xiao-Feng Wang, Email: xf_wang@jlu.edu.cn

Hideki Hashimoto, Email: hideki-hassy@kwansei.ac.jp

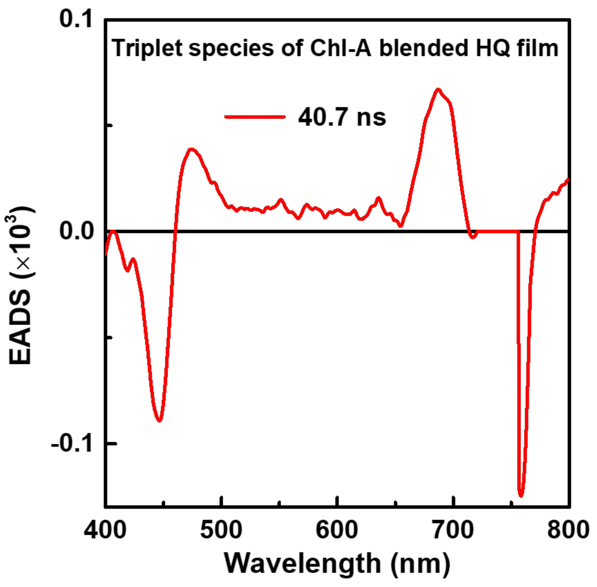

Figure S1. The band shape and lifetime of the triplet-species of Chl-A film blended with HQ pumped at 737 nm.

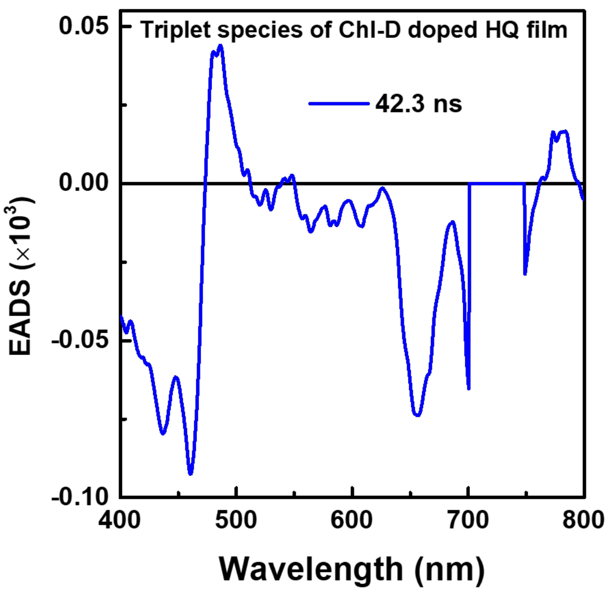

Figure S2. The band shape and lifetime of the triplet Chl-D film blended with HQ pumped at 727 nm.

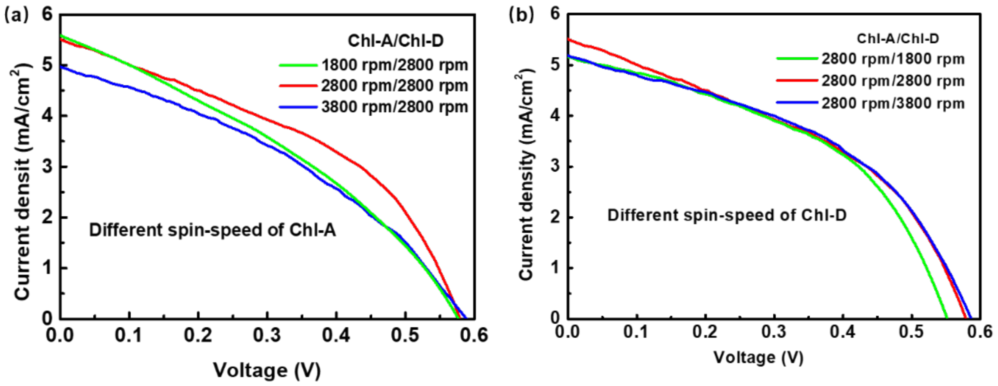

Figure S3. The influence of the film thickness of Chl-A and Chl-D to the final photovoltaic performance of the Z-scheme photosynthesis inspired devices.

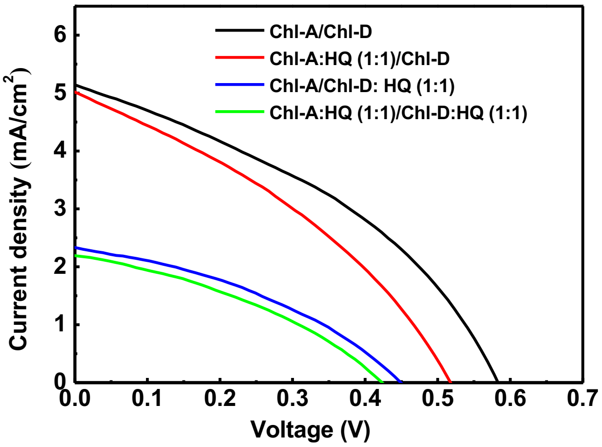

Figure S4. The Photovoltaic performances of the device when the HQ is blended to Chl-A and/or Chl-D with a molar ratio of 1:1, which is the same as the TAS measurement of the film samples.

Table S1. Photovoltaic performances of the Chl-derivatives based bio-solar cells when HQ is blended to Chl-A or/and Chl-D with a molar ratio of 1:1.

Device types	J_{sc} (mA·cm^{-2})	V_{oc} (V)	FF	PCE (%)
Pristine device	5.07	0.58	0.40	1.18
Chl-A:HQ=1:1	4.96	0.51	0.36	0.91
Chl-D:HQ=1:1	2.63	0.44	0.40	0.46
Both blended as 1:1	2.18	0.42	0.40	0.37

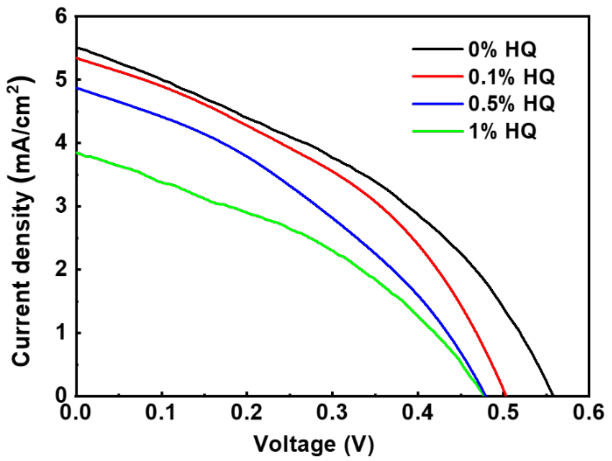

Figure S5. The *J-V* curves of different ratios of HQ doped Chl-D layer-based devices.

Table S2. The photovoltaic performances of the HQ doped Chl-D layer-based bio-solar cells.

Device types	J_{sc} (mA·cm^{-2})	V_{oc} (V)	FF	PCE (%)
0% HQ	5.45	0.56	0.41	1.25
0.1% HQ	5.31	0.50	0.42	1.11
0.5% HQ	4.74	0.47	0.41	0.91
1% HQ	3.93	0.47	0.40	0.74

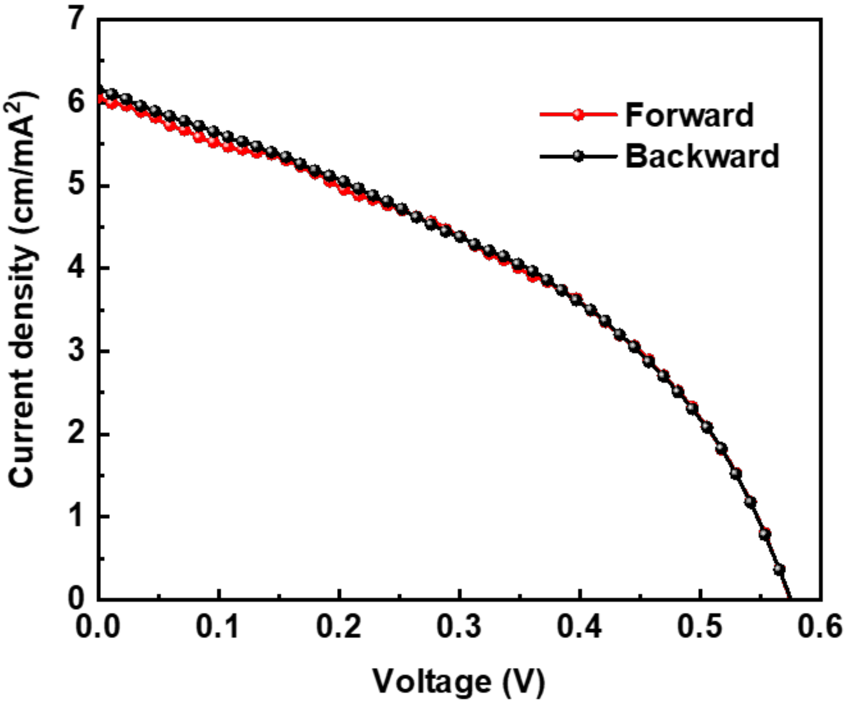

Figure S6. The photovoltaic performance of the 0.5% HQ doped device under forward and backward scan direction.
