# Supplementary material for: Hydroquinone redox mediator enhances the photovoltaic performances of chlorophyll-based bio-inspired solar cells
Source: Commun Chem. 2021 Aug 11;4:118. doi: 10.1038/s42004-021-00556-5 (PMC9814249; doi:10.1038/s42004-021-00556-5)
Supplement: Supplementary file 2 — Solar Cells Reporting Summary [file 42004_2021_556_MOESM2_ESM.pdf]

nature research

Corresponding author(s): *Double-blind peer review submissions: write DBPR and your manuscript number here instead of author names.*

Solar Cells Reporting Summary

Nature Research wishes to improve the reproducibility of the work that we publish. This form is intended for publication with all accepted papers reporting the characterization of photovoltaic devices and provides structure for consistency and transparency in reporting. Some list items might not apply to an individual manuscript, but all fields must be completed for clarity.

For further information on Nature Research policies, including our data availability policy, see Authors & Referees.

Experimental design

Please check: are the following details reported in the manuscript?

1. Dimensions

Item	Yes/No	Details
Area of the tested solar cells	☒ Yes ☐ No	Experimental section on Page 17.
Method used to determine the device area	☒ Yes ☐ No	Experimental section on Page 17.

2. Current-voltage characterization

Item	Yes/No	Details
Current density-voltage (J-V) plots in both forward and backward direction	☒ Yes ☐ No	Figure S4 in the Supporting information.
Voltage scan conditions<br>*For instance: scan direction, speed, dwell times*	☒ Yes ☐ No	Experimental section on Page 17.
Test environment<br>*For instance: characterization temperature, in air or in glove box*	☒ Yes ☐ No	Experimental section on Page 17.
Protocol for preconditioning of the device before its characterization	☒ Yes ☐ No	Experimental section on Page 17.
Stability of the J-V characteristic<br>*Verified with time evolution of the maximum power point or with the photocurrent at maximum power point; see ref. 7 for details.*	☐ Yes ☒ No	The photovoltanic performance comparison of the device is conduced to check whether our conclusion is right or not. Therefore, the stability is not so important in this study.

3. Hysteresis or any other unusual behaviour

Item	Yes/No	Details
Description of the unusual behaviour observed during the characterization	☐ Yes ☒ No	There is no unusual behavior.
Related experimental data	☐ Yes ☒ No	There is no unusual behavior.

4. Efficiency

Item	Yes/No	Details
External quantum efficiency (EQE) or incident photons to current efficiency (IPCE)	☒ Yes ☐ No	On Figure 6b.
A comparison between the integrated response under the standard reference spectrum and the response measure under the simulator	☒ Yes ☐ No	Experimental section on Page 17.
For tandem solar cells, the bias illumination and bias voltage used for each subcell	☐ Yes ☒ No	Our solar cells are not tandem cells.

5. Calibration

Item	Yes/No	Details
Light source and reference cell or sensor used for the characterization	☒ Yes ☐ No	Experimental section on Page 17.
Confirmation that the reference cell was calibrated and certified	☒ Yes ☐ No	Experimental section on Page 17.

Calculation of spectral mismatch between the reference cell and the devices under test

- [ ] Yes
- [x] No

The reference cell and devices are spectrally matched.

6. Mask/aperture

Size of the mask/aperture used during testing

- [x] Yes
- [ ] No

Experimental section on Page 17.

Variation of the measured short-circuit current density with the mask/aperture area

- [ ] Yes
- [x] No

This is not the key point of our study.

7. Performance certification

Identity of the independent certification laboratory that confirmed the photovoltaic performance

- [ ] Yes
- [x] No

We have not conducted the performance certification.

A copy of any certificate(s)
Provide in Supplementary Information

- [ ] Yes
- [x] No

We have not conducted the performance certification.

8. Statistics

Number of solar cells tested

- [x] Yes
- [ ] No

Experimental section on Page 17.

Statistical analysis of the device performance

- [ ] Yes
- [x] No

The photovoltaic performance difference of each device is negligible.

9. Long-term stability analysis

Type of analysis, bias conditions and environmental conditions
For instance: illumination type, temperature, atmosphere humidity, encapsulation method, preconditioning temperature

- [ ] Yes
- [x] No

This is not the key point of our study.
